# Discordance between IHC/FISH and RNA expression-based assessment of ER/HER2 status, and its prognostic impact assessed by propensity score matching

**DOI:** 10.1186/s12885-026-16081-4

**Published:** 2026-05-09

**Authors:** Chiaki Nakauchi, Nanae Masunaga, Yuki Eguchi, Tomonori Tanei, Kenzo Shimazu

**Affiliations:** 1 Department of Breast Surgery, ISEIKAI International General Hospital, 4-14 Minamioogimachi, Kita-Ku, Osaka, 530-0052 Japan; 2 Department of Breast and Endocrine Surgery, Graduate School of Medicine, Osaka University, 2-2-E10 Yamadaoka, Suita, Osaka 565-0871 Japan

**Keywords:** Breast cancer, DNA microarray, Immunohistochemistry, Propensity score matching

## Abstract

**Background:**

Immunohistochemistry (IHC) is widely used to determine estrogen receptor (ER) and human epidermal growth factor receptor 2 (HER2) status, which are crucial for predicting breast cancer recurrence and guiding adjuvant therapy decisions. While RNA expression levels, assessed by microarray, can also determine ER and HER2 status, they do not match IHC assessment results perfectly. This study investigated the discordance rates between IHC and RNA expression-based assessment of ER and HER2 status and evaluated the prognostic impact of such discrepancies.

**Methods:**

IHC/FISH results for determining ER/HER2 status were compared with RNA expression-based assessment. Recurrence-free survival (RFS) analysis was performed to evaluate the association between discordant results and clinical outcomes. Inverse Probability of Treatment Weighting (IPTW) was applied to adjust for potential confounding by differences in patient characteristics. Multivariate analysis was conducted to identify factors most strongly associated with ER/HER2 discordance.

**Results:**

The discordance rate was 11.8% for ER and 19.4% for HER2 when comparing IHC/FISH with RNA expression-based assessment. Analysis of RFS suggested a possible association between discordant ER results and shorter RFS, although this did not reach statistical significance in unadjusted analysis. Using IPTW, discordance in both ER and HER2 status was found to be significantly related to worse RFS (*p* = 0.001 and *p* = 0.04, respectively). In multivariate analysis, PR-negative/HER2-positive cases were most strongly associated with ER/HER2 discordance.

**Conclusions:**

ER and HER2 RNA expression levels should be considered for confirmation in patients with PR-negative/HER2-positive status, as discordance between IHC and RNA-based assessment is significantly associated with worse recurrence-free survival outcomes.

## Introduction

In predicting the prognosis of breast cancer and determining the appropriateness of postoperative adjuvant therapy, the assessment of estrogen receptor (ER) and human epidermal growth factor receptor 2 (HER2) status is crucial. For ER, this is achieved by immunohistochemistry (IHC), while HER2 status is assessed based on the American Society of Clinical Oncology (ASCO)/College of American Pathologists (CAP) guidelines, using both IHC and FISH [1–3].

Since 2000, breast cancer subtype classification using RNA expression levels determined through microarray analysis has been conducted [4], and it has been shown to be more accurate in predicting prognosis than conventional classifications using IHC and FISH [5–8]. Nevertheless, the subtypes classified by microarray do not fully align with the conventional classifications based on IHC and FISH [6].

This study analyses the concordance rate between microarray-based RNA expression levels and the results of IHC and IHC/FISH tests for ER and HER2. The classification of breast cancer has recently undergone significant changes with the emergence of the HER2-low category. Breast cancers were conventionally broadly categorized as HER2-positive or HER2-negative based on immunohistochemistry (IHC) and in situ hybridization (ISH). However, the recognition of the HER2-low category, defined as IHC 1 + or IHC 2 +/ISH-negative, highlights a substantial subgroup previously considered HER2-negative that may now benefit from novel HER2-targeted therapies, such as antibody–drug conjugates (ADCs). Understanding this new category from the perspective of RNA expression offers critical insights into its distinct biological characteristics and potential therapeutic vulnerabilities.

We also hypothesized that the above-mentioned discrepancy impacts prognosis, given that RNA expression levels more precisely characterize tumor subtypes. We thus investigated whether having a discordant result between the conventional IHC/FISH status of ER/HER2 and the determinations of ER/HER2 RNA expression levels using a microarray affects the recurrence-free survival (RFS) rate.

## Materials and methods

### Patients

A total of 314 primary breast cancer patients (stages I–III) who had undergone mastectomy or breast-conserving surgery at Osaka University Hospital between January 1996 and November 2010 were retrospectively included in this study, including 199 cases with known HER2 IHC results and 115 cases with known HER2 FISH results. Written informed consent was obtained from all patients before surgery. The study protocols were approved by the Ethics Committee of Osaka University Graduate School of Medicine (approval number: 11337, date of approval: May 7, 2012). We examined the concordance rate between the conventional method and the microarray-based determinations of ER and HER2. The characteristics of the 314 patients are shown in Table 1.

**Table 1:** Clinicopathological characteristics of 314 patients

	all	ER IHC vs. RNA level		*P*-value	Her2 IHC/FISH vs. RNA level		*P*-value
		concordant	discordant		concordant	discordant	
	(*n* = 314)	(*n* = 277)	(*n* = 37)		(*n* = 253)	(*n* = 61)	
Age(year)							
< = 50	134	121	13	0.3235	110	24	0.5579
> 50	180	156	24		143	37	
Tumor diameter							
< = 20 mm	104	93	11	0.6407	88	16	0.2026
> 20 mm	210	184	26		165	45	
Node metastasis							
Negative	147	124	23	0.0464*	122	25	0.3092
Positive	167	153	14		131	36	
HG							
1 + 2	262	236	26	0.0352*	210	52	0.5665
3	49	39	10		41	8	
ER							
Negative	70	52	18	< 0.0001*	58	12	0.5838
Positive	244	225	19		195	49	
PR							
Negative	138	108	30	< 0.0001*	108	30	0.3591
Positive	176	169	7		145	31	
HER2							
Negative	236	216	20	0.0016*	197	39	0.0238*
Positive	78	61	17		56	22	
Ki67							
< 20%	185	167	18	0.1526	145	40	0.28
≧20%	126	107	19		105	21	

*HG* Histological grade, *ER* Estrogen receptor, *PR* Progesterone receptor, *HER2* Human epidermal growth factor receptor 2

*significant

Among the 314 patients, prognostic analysis was conducted on 190 cases, for whom data on background factors related to prognosis necessary for propensity score matching (age, tumor diameter, nodal status, histological grade, ER status, PR status, HER2 status, Ki67 percentage) were available. These baseline characteristics, including the presence or absence of adjuvant chemotherapy and the surgical decade, which are important prognostic factors, are shown in Table 2. Notably, none of the patients in either group received adjuvant anti-HER2 therapy, as trastuzumab was not approved for adjuvant therapy in Japan until February 2008.

**Table 2:** Clinicopathological characteristics of 190 patients included in the prognostic analysis

	all	ER IHC vs. RNA level		*P*-value	HER2 IHC/FISH vs. RNA level		*P*-value
		concordant	discordant		concordant	discordant	
	(*n* = 190)	(*n* = 168)	(*n* = 22)		(*n* = 166)	(*n* = 24)	
Age(year)							
< = 50	71	66	5	0.1311	63	8	0.6620
> 50	119	102	17		103	16	
Year of surgery							
< = 2000	58	52	6	0.7221	48	10	0.2048
> = 2001	132	116	16		118	14	
Adjuvant chemotherapy							
none	124	110	14	0.9138	109	15	0.7315
done	65	58	7		56	9	
unknown	1	0	1		1	0	
Tumor diameter							
< = 20 mm	92	82	10	0.7672	81	11	0.7861
> 20 mm	98	86	12		85	13	
Node metastasis							
Negative	116	99	17	0.0971	103	13	0.4592
Positive	74	69	5		63	11	
HG							
1 + 2	162	145	17	0.2608	142	20	0.7754
3	28	23	5		24	4	
ER							
Negative	25	13	12	< 0.0001*	23	2	0.4544
Positive	165	155	10		143	22	
PR							
Negative	67	48	19	< 0.0001*	57	10	0.4824
Positive	123	120	3		109	14	
HER2							
Negative	148	136	12	0.0050*	141	7	< 0.0001*
Positive	42	32	10		25	17	
Ki67							
< 20%	130	117	13	0.3167	110	20	0.0927
≧20%	60	51	9		56	4	

*HG* Histological grade, *ER* Estrogen receptor, *PR* Progesterone receptor, *HER2* Human epidermal growth factor receptor

*significant

### RNA extraction and DNA microarray analysis

Tumor tissues were obtained at the time of surgery, immediately snap-frozen in liquid nitrogen, and kept at − 80 °C until RNA extraction. The Qiagen RNeasy® Mini Kit (QIAGEN Science, Germantown, MD, USA) was used to extract RNA from tumor tissues. RNA (200 ng) was subjected to DNA microarray assay (U133 Plus 2.0 Array; Affymetrix, Santa Clara, CA, USA) following the manufacturer’s instructions. Gene Profiling Reagents (Affymetrix) and One Cycle Target Labeling and One Cycle Target Labeling and Control Reagents (Affymetrix) were used.

### Immunohistochemical (IHC) assay

Positivity for ER and PR expression was defined when 10% or more of the tumor cells were stained immunohistochemically (ER: clone 6F11; PR: clone 16; Ventana Japan K.K. and SRL Inc., Tokyo, Japan, respectively). Human epidermal growth factor receptor 2 (HER2) status was examined by immunohistochemistry (anti-human c-erb-2 polyclonal antibody; Nichirei Biosciences, Tokyo, Japan) or by fluorescence in situ hybridization (FISH) using the PathVysion HER2 DNA probe kit (SRL Inc., Tokyo, Japan). For the FISH scoring, the ratio of HER2 gene signals to chromosome 17 signals were calculated for each of the specimens. A tumor that exhibited a +3 immunohistostaining score or a FISH ratio ≥2.0 was considered HER2-positive. The histological grade was determined using the Scarff–Bloom–Richardson grading system. Ki67 was defined as positive when 20% or more of the tumor cells were stained by immunohistochemistry (anti-Ki67 antibody clone 30-9; Roche Applied Science, Mannheim, Germany), as previously described [9].

To address potential technical heterogeneity arising from the prolonged study period (1996–2010), all IHC and FISH assessments were centrally re-evaluated in 2011 at our institution using standardized staining protocols and reagents. Re-assessment was conducted according to the 2011 ASCO/CAP guidelines, ensuring uniform application of classification criteria across all samples. This centralized re-review approach effectively controlled for pre-analytical variability related to fixation methods, antibody reagents, and scoring thresholds.

### Microarray data processing

The gene expression datasets were obtained using a GeneChip™ Human Genome U133 Plus 2.0 Array DNA microarray (Affymetrix). The microarray data is partly available online at the Gene Expression Omnibus website [10] with accession number GSE32646 [11]. ER/HER2 status based on the RNA expression determined by microarray was established using the specific criteria from Recurrence Online tool (https://recurrenceonline.com/gene-array/), which calculates a genomic recurrence score based on the Oncotype DX 21-gene breast cancer assay [12–14]. CEL files were MAS5 normalized, and the log-transformed expression levels of the 16 prognostic genes were calculated relative to five housekeeping genes (ACTB, GAPDH, RPLP0, GUS, and TFRC). ER status was determined using a cutoff value of 500 for probe set 205225_at, while HER2 status was assessed using either bimodal distribution analysis (cutoff: 4,800) or immunohistochemistry-equivalent cutoff (cutoff: 1,150) based on the primary receptor assessment method for probe set 216836_s_at [15–19]. In the absence of a clearly designated probe for PR and Ki67 classification, the mean expression values of all probes targeting these genes were calculated (PR: 208305_at, 208304_at; Ki67: 212020_s_at, 212022_s_at, 212023_s_at).

### Statistical analysis

The statistical software R version 4.4.2 (http://www.R-project.org/), Bioconductor packages (http://www.bioconductor.org/), and JMP software were used for statistical analyses. The associations between the various clinicopathological parameters were evaluated by chi square or Fisher’s exact test. Univariate and multivariate analyses of the various parameters potentially predictive of recurrence and death were conducted with the Cox proportional hazards model. We used the R package “WeightIt” when performing Inverse Probability of Treatment Weighting (IPTW) among the available propensity score matching approaches [20–22].

## Results

### Comparison of the results of ER IHC and HER2 IHC/FISH tests with microarray-based RNA expression levels

**Fig. 1:**
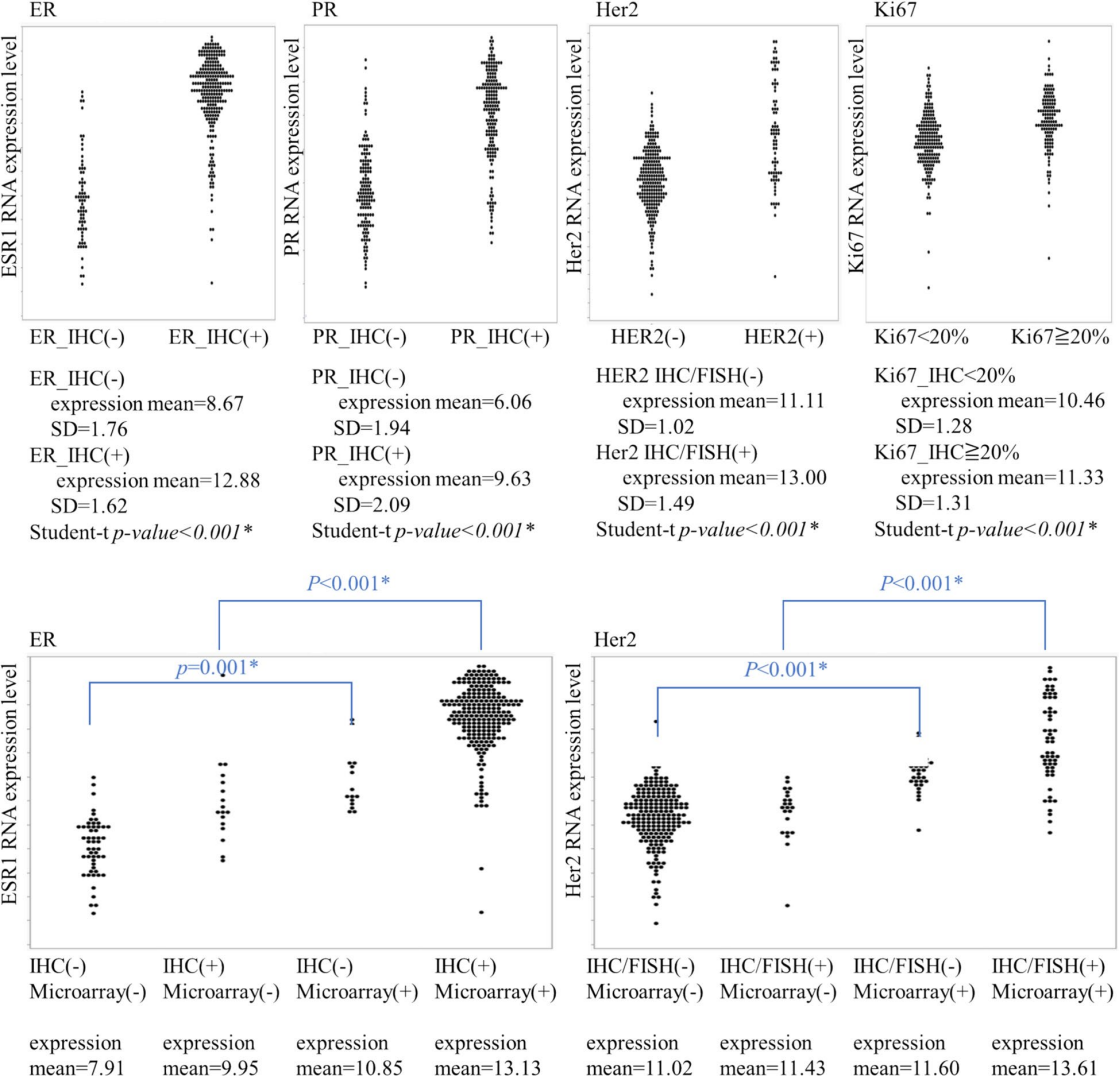
Comparisons of conventional method results with microarray-based RNA expression levels. ER, estrogen receptor; PR, progesterone receptor; HER2, human epidermal growth factor receptor 2; IHC, immunohistochemistry; FISH, fluorescence in situ hybridization; SD, standard deviation

We examined the correlation between the results of ER IHC and HER2 IHC/FISH tests (conventional methods) and the results of the RNA expression levels of ESR1, PR, HER2, and Ki67 determined using a microarray in 314 primary breast cancer patients. Microarray RNA expression levels of ESR1, PR, HER2, and Ki67 were significantly higher in the patient groups conventionally positive for ER, PR, HER2, and Ki67 than in the corresponding negative groups, respectively (all *p*<0.001). Furthermore, coefficients of variation revealed that the conventionally negative groups exhibited less variability across all measured parameters than the conventionally positive groups. Although a correlation was observed between the results obtained by the conventional methods and RNA expression levels, the distributions of microarray-based RNA expression levels for the negative and positive groups partially overlapped (Fig. 1).

In the group classified as ER-positive by conventional methods, those with ER-positive classification by microarray analysis showed significantly higher RNA expression levels compared to those with ER-negative classification by microarray analysis. Similarly, in the group classified as ER-negative by conventional methods, the ER-positive microarray group demonstrated higher RNA expression levels than the ER-negative microarray group. These findings indicate that the microarray-based classification reflects the actual expression intensity of ER. Comparable patterns were observed for HER2, where the microarray-based classification corresponded to the distribution of HER2 expression levels (Fig. 1).

**Fig. 2:**
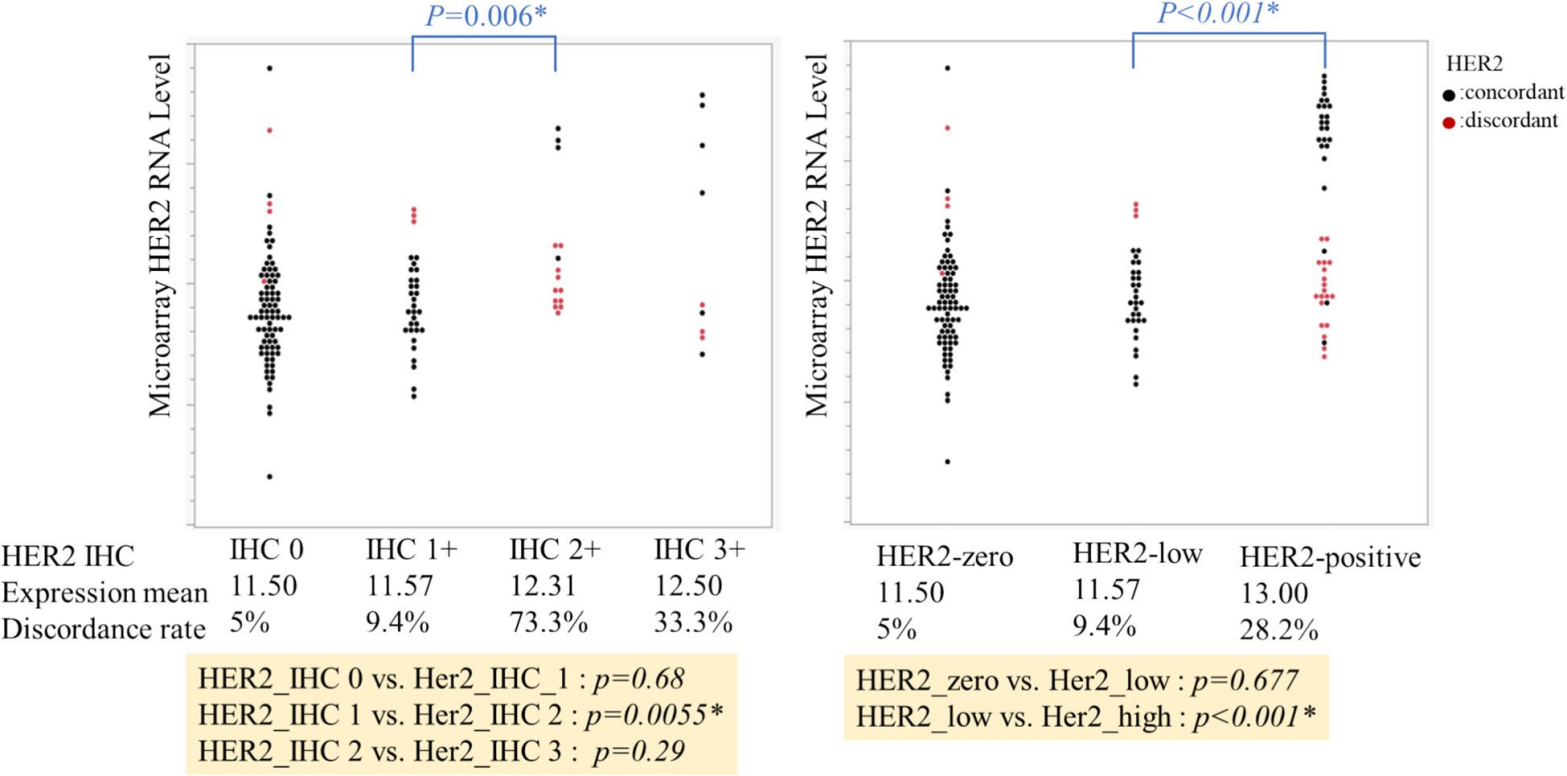
HER2 IHC + FISH conventional results vs. HER2 microarray-based RNA expression levels

Regarding the relevance of HER2 status in the context of breast cancer, growing interest has been generated in the newly recognized HER2-low group. In this study, a significant difference in HER2 RNA expression as determined by microarray was observed only between the HER2 IHC 1+ and HER2 IHC 2+ groups (*p*=0.006), but not between the HER2 IHC 0 and HER2 IHC 1+ groups (*p*=0.68) (Fig. 2). The HER2 IHC 2+ group shows a markedly higher discordance rate (73.3%) compared to other IHC classifications (5–33.3.3% for IHC 0, 1+, and 3+), indicating substantial disagreement between conventional IHC scoring and microarray-based HER2 RNA expression assessment in this intermediate-positive group.

Additionally, we compared the RNA expression levels of HER2 determined using a microarray across three groups: HER2-negative, HER2-low, and HER2-positive. While no significant difference was observed between the HER2-negative and HER2-low groups (*p*=0.677), a significant difference was found between the HER2-low and HER2-positive groups (*p*<0.001) (Fig. 2).

### Concordance rate between RNA expression levels determined using microarrays and the results of ER IHC and HER2 IHC/FISH tests

**Fig. 3:**
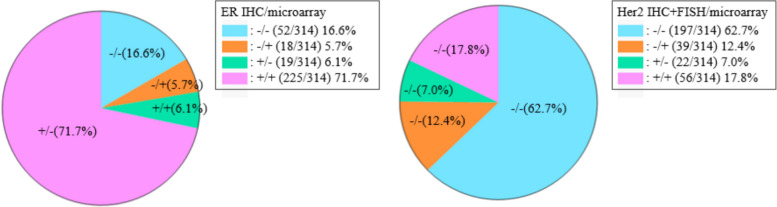
Concordance and discordance of ER/HER2 status: conventional results vs. microarray-based RNA expression

We investigated 314 primary breast cancer patients, including 199 cases with known HER2 immunohistochemistry (IHC) results and 115 cases with known HER2 FISH results. The respective results and correlation coefficients are presented in Table 3 and Fig. 3. A discrepancy rate of 11.8% for ER and 19.4% for HER2 was observed when comparing the conventional ER/HER2 classification with microarray-based determinations (Table 2, Fig. 3).

**Table 3:** Diagnostic accuracy, sensitivity, specificity, PPV, and NPV: conventional results vs. microarray-based RNA expression

ER			HER2		
	Microarray RNA level			Microarray RNA level	
	(-)	(+)		(-)	(+)
IHC(+ FISH)* (-)	52	18	IHC(+ FISH)* (-)	197	39
(+)	19	225	(+)	22	56
Accuracy	88.2%		Accuracy	80.6%	
Sensitivity	92.6%		Sensitivity	58.9%	
Specificity	73.2%		Specificity	90.0%	
PPV	92.2%		PPV	71.8%	
NPV	74.3%		NPV	83.5%	

*ER* Estrogen receptor, *HER2* Human epidermal growth factor receptor 2, *IHC* Immunohistochemistry, *FISH* Fluorescence in situ hybridization

### Prognostic value of the discordance in ER and HER2 status (conventional vs. microarray-based RNA expression) for recurrence-free survival in breast cancer patients

An analysis of RFS was performed on 190 patients, which represented those with complete follow-up data and necessary clinicopathological characteristics from the original cohort of 314 cases evaluated for gene expression. RFS was defined as the time from the date of surgery to the first event of either locoregional recurrence (including ipsilateral breast recurrence, regional lymph node recurrence, or chest wall recurrence), distant metastasis, or death from any cause, whichever occurred first. Patients without any of these events were censored at the last follow-up date.

They were followed up for a median of 75.5 months postoperatively (range, 5–139). Of these 190 patients, 168 were classified as the ER concordant group and 22 patients as the ER discordant group. Moreover, 166 patients were classified as the HER2 concordant group and 24 as the HER2 discordant group. Kaplan–Meier survival analysis suggested that discordant results in ER and HER2 status had a potential impact on RFS, but this did not reach statistical significance.

**Fig. 4:**
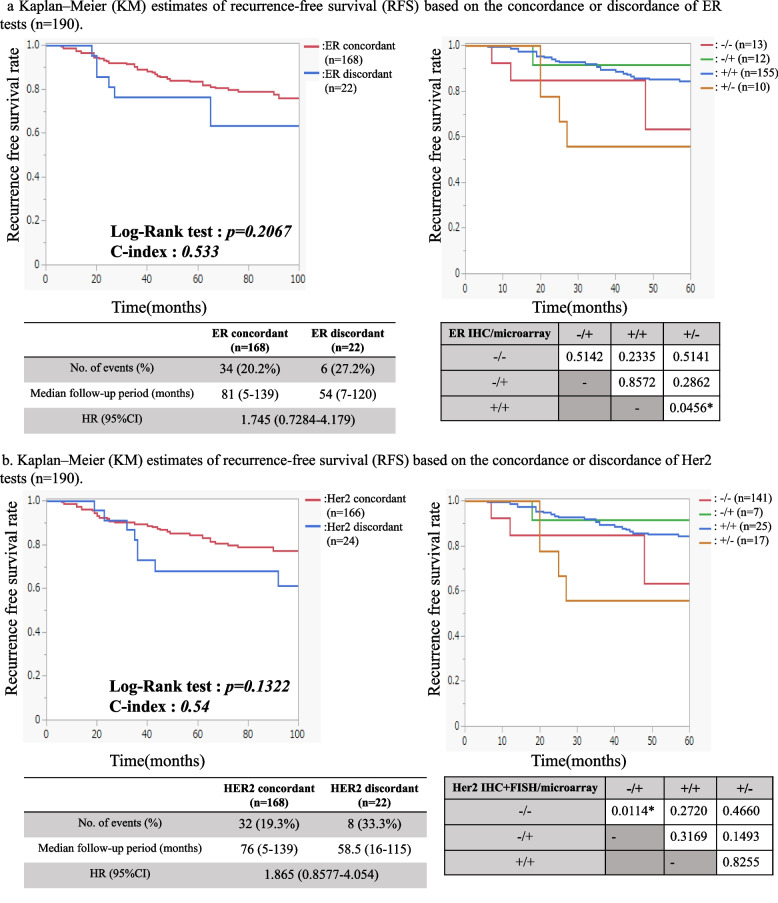
**a** Kaplan–Meier (KM) estimates of recurrence-free survival (RFS) based on the concordance or discordance of ER tests (*n* = 190). **b** Kaplan–Meier (KM) estimates of recurrence-free survival (RFS) based on the concordance or discordance of HER2 tests (*n* = 190)

For ER discordance, the log-rank test yielded a *p*-value of 0.2. Similarly, HER2 discordance demonstrated a p-value of 0.13 (Fig. 4a, b). To assess the predictive accuracy of the survival curves, we calculated the concordance index (C-index) in the ER/HER2 discordant groups (C-index = 0.53 and 0.54, respectively) (Fig. 4a, b). The C-index quantifies the model's discriminative ability to correctly rank survival times between pairs of individuals. However, these C-index values of 0.53 and 0.54 indicated modest discriminative ability, approaching the threshold of random prediction (0.5).

In subgroup analyses, there was a significant difference in RFS of the group with positivity for ER in both the conventional and microarray results when compared with the group with positivity for ER in the conventional results but not in the microarray results (*p* < 0.05) (Fig. 4a). For HER2, there was a significant difference in RFS of the group with positivity for HER2 in both the conventional and microarray results when compared with the group with positivity for HER2 in the conventional results but not in the microarray results (*p* = 0.01) (Fig. 4b). This suggests that patients classified as ER-negative or HER2-positive by conventional methods, but not by microarray analysis, might experience poor prognoses if appropriate adjuvant therapy is not administered. However, we cannot rule out the possibility that confounding factors caused these findings, as cases with discrepancies in ER status (conventional negative/microarray positive) and discrepancies in HER2 status (conventional positive/microarray negative) formed the majority of inconsistent cases.

### Univariate and multivariate analyses to identify factors predictive of concordance/discordance among conventional and microarray RNA ER/HER2 test results

We investigated factors associated with concordance and discordance between conventional and microarray-based ER/HER2 classifications. For ER, multivariate analysis revealed that ER-negative status and PR-negative status were significantly associated with discrepancies between conventional assessment and microarray-based RNA expression levels. Meanwhile, for HER2, HER2-positive status and Ki67 < 20% were significantly associated with discrepancies between conventional assessment and microarray RNA expression levels. Moreover, multivariate analysis showed that a PR-negative status was most significantly associated with discordance in the results for ER independently of the other parameters (*p* < 0.01). Furthermore, multivariate analysis showed that HER2-positive status was most significantly associated with HER2 discordance independently of the other parameters (*p* < 0.0001) (Tables 4 and 5).

**Table 4:** Univariate and multivariate analyses in 190 patients (for ER)

	ER IHC/RNA level discrepancies					
	Univariate analysis			Multivariate analysis		
	Odds ratio	95% CI	*P*-value	Odds ratio	95% CI	*P*-value
Age (years)	2.20	0.77–6.25	0.1311	2.36	0.64–8.74	0.2001
> 50 vs. ≤ 50						
Tumor diameter(mm)	1.14	0.47–2.79	0.7672	1.78	0.57–5.57	0.3222
> 20 vs. ≤ 20						
Node metastasis	0.42	0.15–1.20	0.0971	0.23	0.06–0.82	0.0233
(+) vs. (-)						
HG	1.85	0.62–5.51	0.2608	0.50	0.11–2.22	0.3630
3 vs. 1 + 2						
ER	0.07	0.025–0.19	< 0.0001*	0.18	0.045–0.71	0.0140*
(+) vs. (-)						
PR	0.06	0.02–0.22	< 0.0001*	0.14	0.03–0.61	0.0090*
(+) vs. (-)						
HER2	3.54	1.41–8.92	0.0050*	2.55	0.79–8.23	0.1162
(+) vs. (-)						
Ki67	1.59	0.64–3.95	0.3167	0.98	031–3.12	0.9782
(+) vs. (-)						

*CI* Confidence interval, *HG* Histological grade, *ER* Estrogen receptor, *PR* Progesterone receptor, *HER2* Human epidermal growth factor receptor 2

*significant

**Table 5:** Univariate and multivariate analyses in 190 patients (for HER2)

	HER2 IHC + FISH/RNA level discrepancies					
	Univariate analysis			Multivariate analysis		
	Odds ratio	95% CI	*P*-value	Odds ratio	95% CI	*P*-value
Age (years)	1.22	0.50–3.02	0.6620	1.17	0.34–4.03	0.7978
> 50 vs. ≤ 50						
Tumor diameter(mm)	1.13	0.48–2.66	0.7861	1.51	0.47–4.85	0.4858
> 20 vs. ≤ 20						
Node metastasis	1.38	0.58–3.28	0.4592	1.48	0.45–4.89	0.5161
(+) vs. (-)						
HG	1.18	0.37–3.76	0.7754	1.48	0.45–4.89	0.5161
3 vs. 1 + 2						
ER	1.77	0.29–8.03	0.4544	6.16	0.74–51.4	0.0933
(+) vs. (-)						
PR	0.73	0.31–1.75	0.4824	1.03	0.25–4.32	0.9637
(+) vs. (-)						
HER2	13.7	5.15–36.4	< 0.0001*	48.5	12.2–192.7	< 0.0001*
(+) vs. (-)						
Ki67	0.39	0.13–1.20	0.0927	0.074	0.015–0.37	0.0017*
(+) vs. (-)						

*CI* Confidence interval, *HG* Histological grade, *ER* Estrogen receptor, *PR* Progesterone receptor, *HER2* Human epidermal growth factor receptor 2

*significant

### Kaplan–Meier survival analysis adjusted using Inverse Probability of Treatment Weighting (IPTW)

**Fig. 5:**
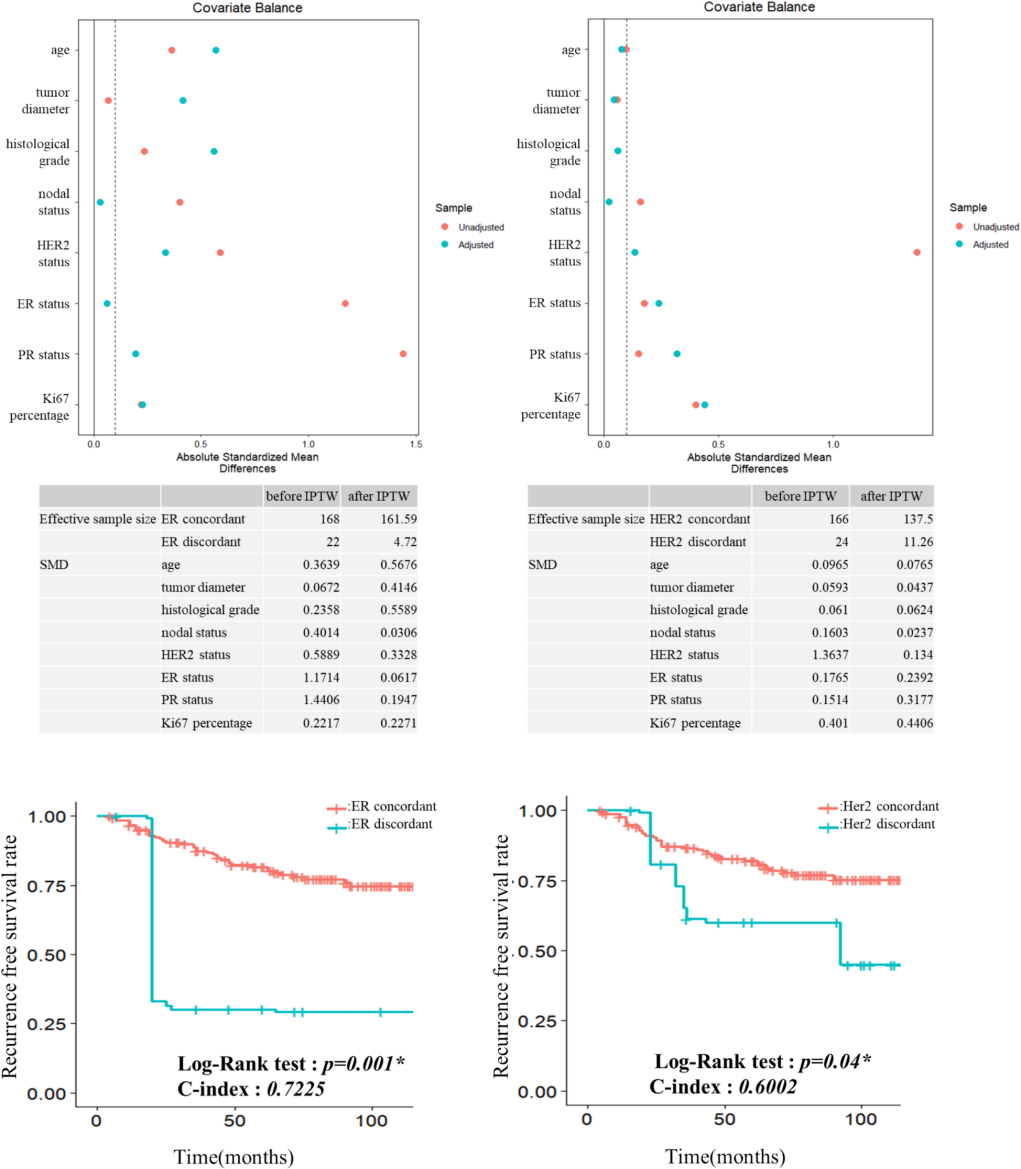
IPTW balance diagnostics and Kaplan–Meier (KM) estimates of recurrence-free survival (RFS) based on the concordance or discordance of ER/HER2 tests (*n* = 190) after adjustment for confounding factors by IPTW

Using Inverse Probability of Treatment Weighting (IPTW), a propensity score matching method, we adjusted for confounding by baseline characteristics to elucidate the pure association of discordance in ER/HER2 IHC and microarray results with prognosis. IPTW was performed to balance the following covariates: age, tumor diameter, nodal status, histological grade, ER status, PR status, HER2 status, and Ki67 percentage. To evaluate the validity of inverse probability of treatment weighting, we calculated standardized mean differences (SMDs) and effective sample sizes before and after IPTW application. Adequate covariate balance was defined as SMD < 0.1 for all covariates. Balance diagnostics were performed for the following covariates: age, tumor diameter, histological grade, nodal status, HER2 status, ER status, PR status, and Ki67 percentage. Balance diagnostics following IPTW weighting are presented in Fig. 5. Substantial improvements in covariate balance were observed across most variables. Notably, HER2 status showed the greatest change in balance (SMD: 1.36 before IPTW → 0.13 after IPTW), and nodal status also demonstrated considerable improvement (SMD: 0.16 → 0.02). Following adjustment, nearly all covariates achieved SMD < 0.1, meeting the criterion for adequate balance. The effective sample size for the ER concordant group decreased from 166 to 137.5 after weighting, while the ER discordant group decreased from 24 to 11.26, which is an expected consequence of the IPTW weighting procedure. These results confirm that the propensity score weighting successfully balanced the treatment groups on observed covariates.

Log-rank test was used for comparisons between groups and Kaplan–Meier analysis revealed significantly poor prognosis in the ER/HER2 discordant groups (*p* = 0.01 and 0.04, respectively) (Fig. 5). Before IPTW, the C-index values were 0.53 and 0.54, indicating limited discrimination. However, after IPTW adjustment, the C-index for ER discordance improved to 0.7225, nearly reaching the 0.8 threshold for strong discrimination, whereas the C-index for HER2 discordance was low at 0.6002 (Fig. 5).

## Discussion

In the present study, we revealed that the discordance between conventional IHC/FISH and microarray-based determinations of ER and HER2 status was associated with a worse prognosis. Specifically, patients with tumors that were ER-positive as determined by conventional methods but ER-negative by microarray analysis exhibited a poorer prognosis. Similarly, those whose tumors were HER2-negative by the conventional assessment but HER2-positive by microarray analysis also showed an unfavorable clinical outcome. These findings suggest that gene expression profiling by microarray analysis provides additional prognostic information beyond conventional IHC/FISH assessment, particularly in discordant cases. The unfavorable prognosis observed in patients with discordant ER/HER2 status likely stems from a critical mismatch between conventional diagnostic assessment and molecular phenotype, leading to suboptimal adjuvant therapeutic decisions. For patients whose tumors were ER-positive by conventional IHC but ER-negative by microarray analysis, the conventional positive result may have led to omission of appropriate adjuvant chemotherapy that would typically be indicated for ER-negative tumors. This therapy-assessment mismatch provides a mechanistic explanation for the poorer clinical outcomes observed in discordant cases.

Regarding the determination of HER2 status, it has been reported that, when conventional methods (IHC with reflex FISH for IHC 2 + equivocal cases) are employed, the false-positive rate is typically less than 6% and the false-negative rate is less than 2%, when compared with the rates when FISH alone is performed on all cases [2, 23, 24]. Compared with these rates, our observed discordance rate of 19.4% between conventional methods and microarray analysis is notably high. This difference suggests that the discrepancies that we identified are not solely attributable to the inherent false-negative or false-positive rates of IHC. Instead, it points to a more complex underlying biological or technical divergence between these two assessment platforms, which warrants further investigation.

Literature data support this observation. Recent studies comparing PAM50 intrinsic subtyping with IHC-based surrogate classification have reported discordance rates of 38–41%, which are notably higher than conventional IHC/FISH concordance rates [6]. For instance, Kim et al. reported 38.4% discordance between IHC-based subtyping and PAM50 intrinsic subtype in 609 breast cancer patients [25]. This substantial discordance between immunohistochemical and transcriptomic assessment methods is increasingly recognized as clinically meaningful, rather than reflecting assay error alone. Our reported discordance rate of 19.4% aligns with the specific focus on individual ER and HER2 gene expression levels (rather than broad intrinsic subtype classification), and suggests platform-specific differences in how these critical biomarkers are assessed.

The observed discordance between conventional IHC/FISH and microarray-based determinations of ER and HER2 status raises the intriguing issue of its underlying cause. It remains unclear whether these differences reflect the microarray’s ability to more comprehensively capture the overall tumor characteristics of heterogeneous tumors, or if the microarray is revealing false negatives or false positives inherent in IHC analyses. Indeed, several factors are known to contribute to potential inaccuracies in IHC. False-positive results can arise from issues such as non-specific antibody binding, endogenous enzyme activity, autofluorescence, and variations in tissue fixation and processing. Conversely, false-negative results may occur due to antigen loss or degradation, or poor antibody quality. Further investigation is needed to elucidate the precise mechanisms behind these discordant findings. Understanding whether these discrepancies stem from the microarray’s superior ability to assess tumor-wide gene expression in heterogeneous contexts, or from the inherent limitations and potential for misclassification by conventional IHC, will be crucial for optimizing diagnostic accuracy and guiding therapeutic decisions [1, 2, 26, 27].

Our multivariate analysis revealed that progesterone receptor (PR) negativity was the strongest independent factor associated with discordant ER status. This aligns with the known biological relationship between ER and PR. When estrogen binds to a functional ER, it triggers intracellular signaling pathways that lead to the induction of PR production [28, 29]. Therefore, the expression of PR is often considered a reliable indicator that the ER signaling pathway is actively functioning. The absence of PR in an otherwise conventionally ER-positive tumor may suggest a dysfunctional ER pathway, potentially explaining the discrepant finding of microarray-based ER negativity and the associated poorer prognosis.

While not all discordant ER cases were PR-negative, our finding that PR-negative tumors are notably prevalent among the discordant ER cases strongly suggests a potential issue with the normal functioning of the ER pathway in these patients. This implies that, even when conventional IHC shows ER positivity, the accompanying PR negativity, as corroborated by microarray-based ER assessment, might indicate a dysfunctional ER signaling cascade. This subtle yet crucial distinction could explain the poorer prognosis observed in these patients, as their tumors may not respond to endocrine therapies as effectively as anticipated based solely on conventional ER positivity.

Multivariate analysis demonstrated that ER discordance was significantly correlated with both conventionally determined ER-negative and PR-negative status. Among cases identified as conventionally ER-negative, 12 of 25 (48%) exhibited ER discordance. Likewise, among those conventionally PR-negative, 19 of 67 (28.4%) were found to be ER-discordant. Multivariate analysis also indicated a significant correlation between HER2 discordance and both conventionally determined HER2-positive status and a low Ki67 proliferative activity (Ki67 < 20%). Among patients conventionally diagnosed as HER2-positive, 17 of 42 (40.5%) exhibited HER2 discordance. Correspondingly, in cases with Ki67 less than 20%, 20 of 130 (15.4%) were found to be HER2-discordant. This suggests that the observed discordance was more prevalent in patients with specific backgrounds, but conventional patient background factors are not sufficient for identifying ER/HER2-discordance.

Given these findings, we propose explicit recommendations for clinical practice. For patients with PR-negative tumors or those with suspected HER2 negativity despite conventional HER2-positive status, reflex RNA-based testing (via RT-qPCR or RNA-Seq analysis of ER and HER2 genes) should be considered. This approach would facilitate identification of discordant cases before treatment selection and enable more informed therapeutic decisions.

While comprehensive microarray analysis (such as Oncotype DX) remains expensive for routine implementation, reviewing existing Oncotype DX results is practical and feasible. In cases where ER/HER2 discrepancy is suspected based on clinical presentation (e.g., PR-negative, ER-positive tumors with poor initial response, or low-proliferation, HER2-positive cases), Oncotype DX results should be revisited. If discrepancy is confirmed, reconsideration of treatment strategy is warranted. Additionally, when patients undergoing or completing adjuvant therapy fail to demonstrate expected therapeutic benefit despite favorable conventional clinicopathological features, measurement of ER and HER2 RNA expression should be considered to identify potential discordant biology.

Moving forward, development of more cost-effective RNA expression systems, including RT-qPCR and RNA-Seq, will be essential for integrating these molecular insights into routine clinical practice. Such approaches would enable broader application of RNA-based validation, particularly as targeted therapies (endocrine agents, HER2-directed therapies) continue to evolve.

## Data Availability

The datasets generated and analyzed during the current study are available from the corresponding author on reasonable request.

## Abbreviations

HG: Histological grade
ER: Estrogen receptor
PR: Progesterone receptor
HER2: Human epidermal growth factor receptor
CI: Confidence interval
